# In Vivo Antimalarial Activity of Leaf Extracts and a Major Compound Isolated from *Ranunculus multifidus* Forsk

**DOI:** 10.3390/molecules26206179

**Published:** 2021-10-13

**Authors:** Betelhem Sirak, Lea Mann, Adrian Richter, Kaleab Asres, Peter Imming

**Affiliations:** 1 Department of Pharmacy, College of Medicine and Health Sciences, Arba Minch University, Arba Minch P.O. Box 21, Ethiopia; betelhem.sirak@amu.edu.et; 2 Department of Pharmaceutical Chemistry, Faculty of Natural Sciences, Martin-Luther-Universitaet Halle-Wittenberg, 3, 06120 Halle (Saale), Germany; lea.mann@pharmazie.uni-halle.de (L.M.); adrian.richter@pharmazie.uni-halle.de (A.R.); 3 Department of Pharmaceutical Chemistry and Pharmacognosy, School of Pharmacy, College of Health Sciences, Addis Ababa University, Addis Ababa P.O. Box 1176, Ethiopia

**Keywords:** *Ranunculus multifidus*, 4-day suppressive test, Rane’s test, prophylactic test, anemonin

## Abstract

The leaves of *Ranunculus multifidus* Forsk. are traditionally used for the treatment of malaria in several African countries. In the present study, 80% methanol (RM-M) and hydrodistilled (RM-H) extracts of fresh leaves from *R. multifidus* and its major constituent anemonin were tested for their in vivo antimalarial activity against *Plasmodium berghei* in mice. Anemonin was also tested for its in vitro antimycobacterial activity against *Mycobacterium smegmatis* and *M. abscessus* in a microbroth dilution assay, and bacterial growth was analyzed by OD measurement. The isolation of anemonin from RM-H was carried out using preparative thin layer chromatography (PTLC). The chemical structures of anemonin and its hydrolysis product were elucidated using spectroscopic methods (HR–MS; 1D and 2D-NMR). Results of the study revealed that both RM-M and RM-H were active against *P. berghei* in mice, although the latter demonstrated superior activity (*p* < 0.001), as compared to the former. At a dose of 35.00 mg/kg/day, RM-H demonstrated a chemosuppression value of 70% in a 4-day suppressive test. In a 4-day suppressive, Rane’s and prophylactic antimalarial tests, anemonin showed median effective doses (ED_50_s) of 2.17, 2.78 and 2.70 μM, respectively. However, anemonin did not inhibit the growth of *M. smegmatis* and *M. abscessus*.

## 1. Introduction

Malaria is one of the most pressing public health issues in developing countries [1]. There were an estimated 229 million malaria cases in 87 malaria-endemic countries worldwide (as of 2019), in which 94% of the cases (215 million) occurred in the African region [1]. The emergence and swift spread of multi-drug resistant *Plasmodium* parasites, the development of insecticide-resistant mosquitos, and the absence of effective vaccines are some of the challenges in the process of controlling and eventually eradicating malaria [2,3]. In Ethiopia, malaria is one of the main public health and economic problems, with 75% of the landscape below 2000 m above sea level affected by the disease [4]. In recent years, Ethiopia achieved a reduction in their malaria mortality rate by more than 40% [1], yet the disease is still one of the leading causes of morbidity and mortality [5].

*Ranunculus multifidus* Forsk. is a perennial herb that grows in moist, open grassy meadows near rivers, streams, and lakes as well as on wet slopes and in open mountain forests and is mostly considered a weed [6]. In the Democratic Republic of Congo, the leaves of *R. multifidus* are used for the treatment of malaria [7] while in different parts of Ethiopia, they are used to treat leishmaniasis [8,9,10]. In South Africa, it is used to treat sexually transmitted diseases, tuberculosis, genital sores, warts, and hemorrhoids [11,12].

Most members of the genus *Ranunculus* biosynthesize the toxic glycoside ranunculin, which is then converted into protoanemonin via enzymatic hydrolysis. Protoanemonin will then cyclodimerize to yield the water-insoluble crystalline dimer anemonin [13]. A previous study demonstrated that the whole plant of *R. multifidus* extracted with dichloromethane–methanol (1:1), possesses in vitro antimalarial activity against a chloroquine-sensitive strain of *Plasmodium falciparum* with an IC_50_ value of 2.3 μg/mL [14]. The objective of this study was to investigate the in vivo antimalarial effect of extracts from the leaves of *R. multifidus* and its major constituent in mice infected with a chloroquine-sensitive strain of *P. berghei*.

## 2. Results and Discussion

### 2.1. Acute Oral Toxicity

The acute toxicity test results of the present study documented that the hydroalcoholic extract (RM-M) was safe by the oral route at a dose of 2000 mg/kg as no mortality or signs of toxicity were observed within 14 days, which suggests that the 50% lethal dose (LD_50_) of RM-M is above 2000 mg/kg. However, at the same dose, the hydrodistilled extract (RM-H) showed severe toxicity leading to death in experimental animals. The main test revealed that RM-H has an LD_50_ higher than 175 mg/kg but lower than 550 mg/kg.

### 2.2. Antimalarial Activity of Extracts

In the current study, the in vivo antimalarial activity of *R. multifidus* leaf extracts was examined using the classic 4-day suppressive test to evaluate the chemosuppressive effect of the plant extract during early infection. The percent of the inhibition of parasitemia (percent of suppression) and the survival time were taken as parameters for the determination of activity of the test substances throughout the assay. Moreover, as malarial infection causes anemia, changes in body (rectal) temperature, and body weight loss, the potential of the tested substances to reduce anemia, prevent body weight loss, and regulate body temperature in the infected mice were also studied.

The experimental results showed that both RM-M and RM-H provide a chemosuppressive effect on parasitemia in a manner that is dose-independent. These effects were statistically significant relative to the negative control group (*p* < 0.001). The chemosuppression percentages ranged from 39.2% to 70.6%. As shown in Table 1, RM-H exhibited a significant inhibition (*p* < 0.001) of parasite multiplication (70.6%) at an oral dose of 35.00 mg/kg per day. All tested doses of the extracts exhibited significantly lower (*p* < 0.001) parasitemia reduction compared to the positive control group. Moreover, the test substances prolonged mean survival time (MST) significantly (*p* < 0.001), as compared to the negative control group; however, the values were significantly (*p* < 0.001) smaller than the standard drug chloroquine.

The 4-day suppressive test results demonstrated that the tested extracts prevent body weight reduction significantly (*p* < 0.001), as compared to the negative control group. While the effects of the lower (100 mg/kg/day) and middle (200 mg/kg/day) doses of RM-M were much less than that of the positive control group, the highest dose of RM-M and all dose levels of RM-H significantly (*p* < 0.001) protected parasite-induced weight reduction in infected mice, as compared to those in the negative control group (Table 2). All dose levels of the tested extracts significantly (*p* < 0.001) prevented a reduction in rectal temperature, as compared to the negative control group. As shown in Table 2, except for the lower (100 mg/kg/day) and middle (200 mg/kg/day) doses of RM-M, all others displayed statistically comparable temperature stabilization effects, as compared with that of the standard drug. All dose levels of RM-M and RM-H exhibited a statistically significant (*p* < 0.001) effect on the circumvention of packed-cell-volume (PCV) decline in *P. berghei*-infected mice, as compared to the negative control group, although their effect was lower (*p* < 0.001) than that of the standard drug.

The analysis of the 4-day suppressive test results indicated that both leaf extracts of *R. multifidus* inhibited parasite multiplication in *P. berghei*-infected mice, confirming the potential of the plant extracts to prevent or mitigate a primary attack due to malaria. Furthermore, the extracts improved survival time, prevented weight loss, maintained rectal temperature, and ameliorated anemia of infected mice, indicating that the plant extracts have the capacity to reduce the overall pathogenic outcome of the parasite on the test groups [15,16]. In the 4-day suppressive test, RM-H showed superior activity (*p* < 0.001), as compared to RM-M.

### 2.3. Structural Elucidation of the Isolated Compound

**Figure 1:**
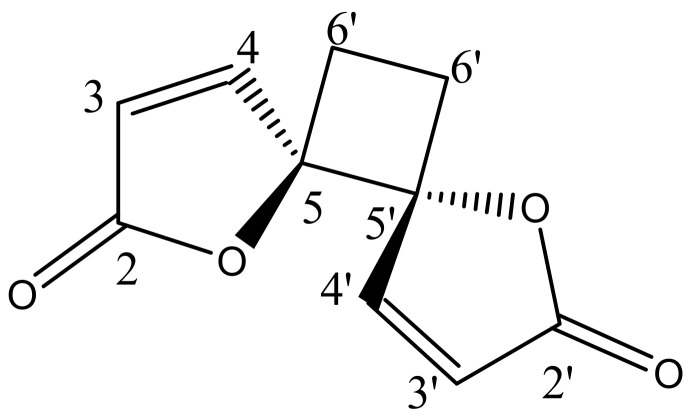
Structural formula of anemonin.

The phytochemical investigation of RM-H using a hexane–ethyl acetate (5:1) solvent system over a silica gel PTLC resulted in the isolation of a pale yellow irritating oil that solidified immediately at room temperature to a white crystalline powder. The white powder was further purified by silica gel PTLC using a mixture of ethyl acetate and hexane in a ratio of 3:2 as a solvent system to yield a compound designated as RM-H1 (R*_f_* = 0.59). The HRMS of RM-H1 showed a pseudo-molecular ion (M+1) peak at 193.0496 amu (calc. *m/z* 193.0495 amu), which corresponds to a relative molecular formula of C_10_H_9_O_4_^+^. The ^1^H-NMR spectrum of RM-H1 revealed the presence of four cyclobutane protons assigned to H-6a, H-6a′ (δ 2.64, 2H, m) and H-6b, H-6b′ (δ 2.25, 2H, m). Four furanone protons were also observed and assigned to H-3, H-3′ (δ 6.32, 2H, d, *J* = 5.7 Hz) and H-4, H-4′ (δ 8.28, 2H, d, *J* = 5.7 Hz). The ^13^C-NMR spectrum of RM-H1 displayed five signals representing 10 carbon atoms, an indication that the compound is a dimer. The DEPT-135 spectrum revealed the presence of two CH_2_ and four methine carbons. The signals at δ 171.8, 156.5, 121.1, and 90.44 were assigned to the lactone carbonyl (C-2 and C-2′), olefinic (C-4 and C-4′), (C-3 and C3′) and methine (C-5 and C-5′) carbons, respectively. The upfield peak at δ 23.8 was assigned to the methylene carbons of the cyclobutane ring at C-6 and C-6′. The ^1^H-^1^H COSY spectrum clearly showed a cross peak between H-3, H-3′ and H-4, H-4′. A further spin-spin coupling between H-6a, H-6a′ and H-6b, H-6b′ is also apparent in the spectrum. The complete assignments of ^1^H and ^13^C chemical shifts of RM-H1 are listed in Table 3. From the chemical shifts presented and by comparing the ^1^H and ^13^C-NMR spectral data of RM-H1 with the compound reported earlier [17], RM-H1 was characterized as anemonin (Figure 1).

### 2.4. Structural Elucidation of the Hydrolysis Product of Anemonin

Two different hydrolysis products of anemonin were reported in the literature: 4,7-dioxo-2-decenedioic acid with no details as to hydrolysis and structure determination [18] and sodium 2-(10*E*) 2′-sodium-carboxylate-vinyl)- 5-oxocyclohex-1-en carboxylate [19]. Since the hydrolysis of anemonin may occur in culture media and will influence activity, 90 mg of the isolated anemonin was subjected to hydrolysis by sodium hydroxide in methanol, following the procedure of Nono et al. [19]. The TLC monitoring showed that the decomposition of anemonin only began at an elevated temperature, as reported [19]. After acidification, extraction, and HPLC, 20 mg of a slightly yellow powder was isolated, which proved to be the acid corresponding to the disodium salt reported by Nono et al. [19]. The exact mass was determined by ESI-MS to be 233.0416 amu (z = 1), which corresponds to the expected mass of the mono sodium salt (calc for C_10_H_10_O_5_Na^+^ [M+H^+^]: 233.0420 amu). ^1^H NMR (CD_3_OD): 6.86 (d, 1H, *J* = 15 Hz), 6.16 (d, 1H, *J* = 15 Hz), 3.21 (s, 2H), 2.88 (m, 2 H), 2.47 (m, 2H). ^13^C NMR (CD_3_OD, DMSO-*d*6): 208.7, 171.7, 167.8, 167.6, 136.0, 133.7, 126.1, 33.6, 28.4, 28.3.

### 2.5. Acute Oral Toxicity of Anemonin

Results of the present study showed that anemonin and RM-H have similar oral acute toxicity profiles. Thus, the LD_50_ of anemonin was determined to be more than 175 mg/kg but less than 550 mg/kg. Anemonin was reported to be toxic to primary keratinocytes above a concentration of 25 μg/mL in in vitro 3-(4,5-dimethylthiazole-2-yl)-2,5-diphenyltetrazolium bromide (MTT) assay [20].

### 2.6. Antimalarial Activity of Anemonin

#### 2.6.1. Four-Day Suppressive Test

The results of the 4-day suppressive test indicated that anemonin significantly reduced parasitemia (*p* < 0.001), as compared to the negative control group, with the percentage of suppression ranging from 78.3% to 83.0% (Table 4 and Supplementary Materials). However, all doses of anemonin exhibited significantly lower (*p* < 0.001) parasitemia reduction, as compared to the positive control group. Moreover, all test substances prolonged the MST significantly (*p* < 0.001), as compared to the negative control group. However, the MST was still significantly smaller (*p* < 0.001) than that of the standard drug chloroquine. Overall, significant statistical differences were not observed among doses of anemonin.

The 4-day suppressive test results showed that anemonin prevented the reduction of body weight significantly (*p* < 0.001), as compared to the negative control group (Table 5). All doses of anemonin significantly prevented (*p* < 0.001) the reduction of body (rectal) temperature due to an infection with *P. berghei*, as compared to the negative control group. Anemonin also displayed statistically comparable effects with the standard drug in temperature stabilization. Anemonin showed protection of RBCs from *P. berghei*-infection-associated anemia (*p* < 0.001), as compared to the negative control group. However, its effect was lower (*p* < 0.001) than that of chloroquine.

#### 2.6.2. Rane’s Test

**Figure 2:**
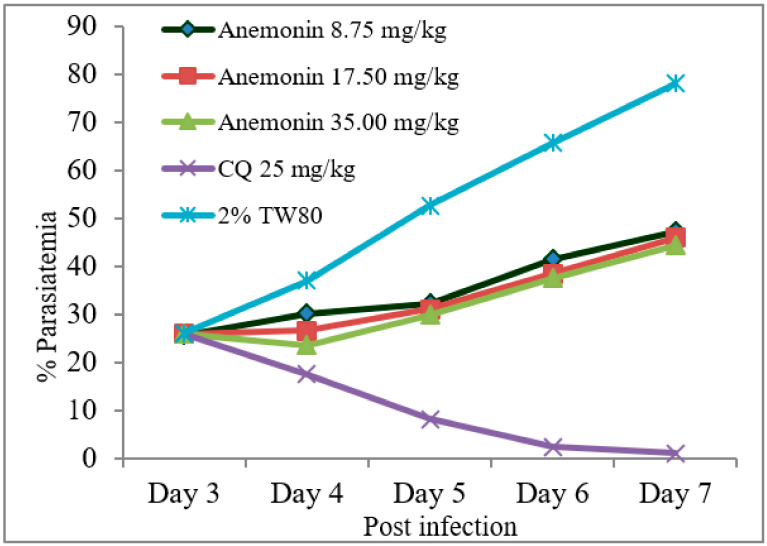
Parasitemia development over the course of treatment with anemonin on *Plasmodium berghei*-infected mice in Rane’s antimalarial test (CQ; chloroquine, 2% TW80: 2% Tween 80).

As shown in Figure 2, Rane’s test resulted in a gradual escalation of parasitemia throughout the course of treatment in the anemonin-treated groups (Figure 2). A repeated measures two-way ANOVA analysis of parasitemia showed a significant (*p* < 0.001) difference in parasite development across the course of treatment. At all dose levels, anemonin treatment resulted in a significant (*p* < 0.001) reduction of parasitemia, as compared to the negative control group, but the effect was less than the positive control group (Table 6 and Supplementary Materials). The efficacy of anemonin in mice at all doses was correlated significantly (*p* < 0.001) with an increased MST, as compared to the untreated control animals. However, increases in the MST of the chloroquine-treated group was significantly (*p* < 0.001) higher than that of anemonin-treated group.

All the tested doses of anemonin protected against body weight loss significantly (*p* < 0.001), as compared to the negative control group, but the effect of chloroquine was superior (*p* < 0.001) to that of anemonin. Similarly, anemonin prevented the reduction of body temperature in the mice, and the effect was statistically significant (*p* < 0.001), as compared to the negative control group. There was no statistical difference between anemonin, at all dose levels, and chloroquine on improving body temperatures of the mice. As shown in Table 7, when anemonin was administered to *P. berghei*-infected mice, a substantial improvement of the PCV was observed, as compared with the infected, untreated group. Overall, in established infections of *P. berghei*, anemonin provided significant suppression of parasitemia and maintained pathological parameters, which suggests a curative potential for the compound.

#### 2.6.3. Prophylactic Test

In the prophylactic test, all the tested doses of anemonin significantly (*p* < 0.001) suppressed parasitemia, as compared to the negative control group (Table 8). However, the effect of anemonin was significantly (*p* < 0.001) lower than the chemosuppression displayed by the positive control group. Similarly, anemonin caused significant (*p <* 0.001) prolongation of the MST, as compared to the negative control groups, although chloroquine was more effective (*p <* 0.001) than anemonin (Table 8 and Supplementary Materials).

Treatment with anemonin significantly prevented body weight loss associated with parasitemia at all dose levels on day 7, as compared to body weight on day 3. The results also revealed that anemonin-treated groups at all dose levels significantly (*p* < 0.001) prevented the loss of body weight, as compared to that of the negative control group. Similarly, anemonin significantly attenuated (*p* < 0.001) the rapid decline in rectal temperature of infected mice, as compared to the negative control group, and there were no statistical differences between the effects of anemonin and chloroquine. In addition, anemonin prevented the reduction of PCV significantly (*p* < 0.001) due to the parasite infection, as compared with the negative control group. The effect of a higher dose of anemonin (35.00 mg/kg/day) was comparable with that of chloroquine (Table 9).

An increase in parasitemia levels in rodents usually results in decreased metabolic rates, and they develop severe hypothermia [15], which may result in death. The decrease in body weight caused by malaria has been associated with decreased food intake, disturbed metabolic function, and hypoglycemia [15]. An ideal antimalarial agent would, therefore, prevent this occurrence. In the current study, anemonin showed temperature stabilizing and weight maintenance effects in all models. In addition to parasite suppression, this may indicate that anemonin controlled the immune system of the infected mice as well as controlled some of the pathological processes and balanced the reduction of the metabolic rate that produces the drop in rectal temperature.

**Figure 3:**
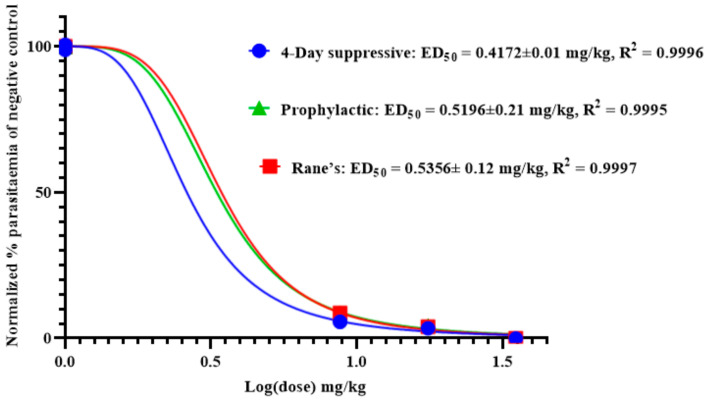
ED_50_ of anemonin in mice infected with *Plasmodium berghei* in 4-day suppressive, Rane’s, and prophylactic antimalarial tests; ED_50_ was estimated from a plot of log dose against the percentage of parasitemia of negative control group (normalized); values are presented as mean ± SEM; *n* = 5.

As shown in Figure 3, the effective median dose (ED_50_) of anemonin was determined by a non-linear regression analysis from sigmoidal dose–response curves using Graphpad Prism 8.0 software. This revealed that the ED_50_s of anemonin are 0.4172, 0.5356, and 0.5196 mg/kg (2.17, 2.78, and 2.70 μM) in the 4-day suppressive, Rane’s, and prophylactic tests, respectively.

Although there is an ongoing debate about the cut-off value for the potency of natural products, the industry standard for considering a pure compound to be active is generally accepted as IC_50_ ≤ 10 µM [21]. In this regard, anemonin can be considered a potent antimalarial compound with the potential to be developed into a viable antimalarial drug. Therefore, the use of anemonin as a template for designing novel antiplasmodial pharmacophores cannot be overemphasized.

The trophozoite stage of the malarial parasite in human erythrocytes exhibits an intense glutathione (GSH) metabolism, and many glutathione species occur in trophozoite cytosol, mainly GSH [22]. GSH is a water-soluble tripeptide composed of the amino acids glutamine, cysteine, and glycine, and it plays an important role as an antioxidative defense (scavenging free radicals generated by the parasite) as well as maintaining an environment to reduce cytosol, promoting rapid cell growth, and supporting many of the known GSH-dependent processes that are directly related to the specific lifestyle of the parasite [22,23]. All these are considered factors in the pathophysiology of malaria but also as potential drug targets [24,25]. Methylene blue, an inhibitor of the structurally known *P. falciparum* glutathione reductase, appears to be a promising antimalarial medication when given in combination with chloroquine [26,27]. Cysteine thiol acts as a nucleophile in reactions with both exogenous and endogenous electrophilic species [28,29]. As a consequence, reactive oxygen species are frequently targeted by GSH in both spontaneous and catalytic reactions [22,30]. Compounds containing an α,β-unsaturated lactone, such as anemonin, undergo a specific alkylation involving unsaturated lactone with the thiol group (sulphydryl (-SH)) of L-cysteine (2) by a Michael-type addition [31,32,33].

**Figure 4:**
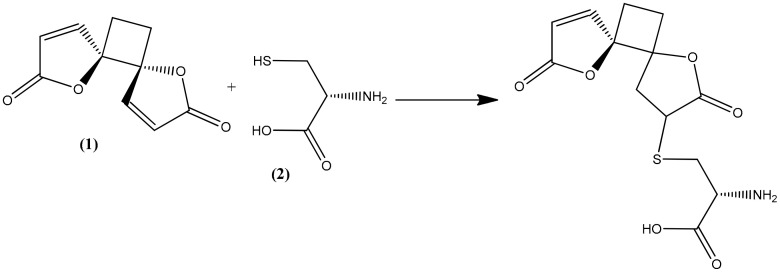
Possible Michael-type addition reaction between anemonin (1) and L-cysteine (2).

In the present study, the observed antiprotozoal activity of anemonin might be by increasing oxidative stress in the parasite, since it interacts with the thiol group of the precursor amino acid L-cysteine, along with the cysteine residue of GSH itself by a Michael-type addition (Figure 4). Meanwhile, L-cysteine is relevant as a substrate in the synthesis of GSH [34]. The capability of anemonin to interact with this important amino acid may suggest its potential to increase oxidative stress and inhibit growth in the parasites.

### 2.7. Antimycobacterial Activity of Anemonin

Since *R. multifidus* is also traditionally used for the treatment of tuberculosis and tuberculosis-like lung infections, anemonin was tested for in vitro activity against two mycobacterial species: viz. *Mycobacterium smegmatis*, which is a good model for *M. tuberculosis*, and the fast-growing *M. abscessus*, which is an emergent health threat, especially for people with lung problems. The antimycobacterial activity was determined against *M. abscessus* ATCC 19977 and *M. smegmatis* mc^2^ 155 [35]. Even with a concentration up to 100 µM, anemonin showed no growth inhibition in either fast-growing mycobacteria.

## 3. Materials and Methods

### 3.1. Plant Material

Fresh leaves of *R. multifidus* were collected from Dorze, a village located in Chencha woreda and part of the Gamo Gofa Zone (520 km southwest of Addis Ababa, Ethiopia), which is located in the Great Rift Valley, above the west shore of Lake Abaya, at 6°11′36″ N and 37°34′13″ E. The plant material was authenticated by Ato Melaku Wondafrash, National Herbarium, Department of Biology, College of Natural and Computational Sciences, Addis Ababa University (AAU), where a botanical specimen was deposited (collection number BS-001) for future reference.

### 3.2. Preparation of Ranunculus multifidus Leaf Extracts

(1) Aqueous methanol extract, RM-M: Fresh leaves (2 kg) of *R. multifidus* were macerated with about 6 L of 80% methanol at room temperature for 9 days, and the filtrate was concentrated in a rotavapor (Heidolph Instruments GmbH and Co., Schwabach, Germany) at a temperature not exceeding 40 °C. The remaining aqueous solution was dried in a lyophilizer (Alpha 1-2LD plus Martin Christ Co. Ltd., Osterode, Germany) to yield 6.4% (*w*/*w*) of brown powder labeled as RM-M.

(2) Chloroform extract of hydrodistillate, RM-H: Fresh leaves (1 kg) of *R. multifidus* were chopped into small pieces and subjected to hydrodistillation for 3 h using a Clevenger-type apparatus. The condensate was collected and extracted with 100 mL chloroform (3×) and using a separatory funnel. About 5 g of ahydrous sodium sulfate was added to the combined organic solvent extract to remove moisture, and then it was filtered using Whatman No 1. filter paper. The organic solvent was concentrated in a rotavapor at a temperature not exceeding 35 °C to yield 0.56% (*w*/*w*) pungent oil designated as RM-H.

### 3.3. Chemicals

Chromatographic separation was performed by preparative TLC Silica gel 60 F254 (0.5 mm thick) (Loba Chemie Pvt. Ltd., Mumbai, India). *n*-Hexane, chloroform, ethyl acetate and methanol (Loba Chemie Pvt. Ltd., Mumbai, India), trisodium citrate (BDH Chemicals Ltd., London, UK), and Giemsa (ESJAY Chemicals, Maharashtra, India) were all used as received. Pure chloroquine phosphate supplied by Ethiopian Pharmaceuticals Manufacturing Sh. Co. (EPHARM, Addis Ababa, Ethiopia) was used as a reference drug.

### 3.4. Preparative Thin Layer Chromatography

Preparative thin layer chromatography (PTLC) was carried out using a mixture of *n*-hexane and ethyl acetate (5:1) as a mobile phase to analyze RM-H. The chromatograms were visualized using ultraviolet light (UV) at wavelength 254 nm. The major band was carefully scrapped off the plates, washed with a mixture of chloroform and methanol (1:1), and then filtered and concentrated to dryness under reduced pressure. The white powder obtained, designated RM-H1, was further purified by PTLC using ethyl acetate–hexane (3:2) as a solvent system.

### 3.5. Mass Spectrometry

High-resolution mass spectra (HRMS) were recorded on an LTQ Orbitrap XL mass spectrometer (Thermo Fisher Scientific, Bremen, Germany) using nano-ESI (Proxeon, Odense, Denmark), and samples were loaded into self-pulled, gold-coated quartz emitters. Parameters included damping gases helium (purity 5.0, linear trap) and nitrogen (purity 5.0, curved trap). Ionization mode was positive. Mass range was *m*/*z* 100–2000. No sheath and auxiliary gas (nano-ESI), no heated vaporizer, capillary temperature was 200 °C, spray voltage was 1.3 kV, capillary voltage was 43 V. Tube lens voltage was 100 V with max. injection time of 100 ms.

### 3.6. NMR

NMR spectra were obtained at room temperature on a VNMRS 400 MHz spectrometer (Agilent Technologies, Santa Clara, CA, USA) operating at 400 MHz for ^1^H and 100 MHz for ^13^C using deuterated dimethyl sulfoxide (DMSO) as a solvent. Chemical shifts are reported relative to the residual solvent peak of DMSO-d_6_ (^1^H = 2.50 ppm, ^13^C = 39.5 ppm).

### 3.7. Experimental Animals and Parasite

White Swiss albino mice of either sex, weighing 22–30 g and aged 5–6 weeks, were employed throughout the experiment. The mice were obtained from the animal house of the Department of Pharmacology, School of Pharmacy (SoP), College of Health Sciences, AAU. The animals were held in stainless steel cages at room temperature with a 12 h light/12 h dark cycle. They were provided with water and food pellets ad libitum in the animal house. All the experiments were conducted in accordance with internationally accepted laboratory animal use and care guideline [36] and were approved by the Institutional Review Board of SoP, AAU (approval code: ERB/SOP241b/13/2021). The chloroquine-sensitive *Plasmodium berghei* ANKA strain was used for antimalarial assay. The parasites were subsequently maintained in the laboratory by serial blood passage from the infected mice to the non-infected ones on a weekly basis.

### 3.8. Acute Oral Toxicity

An acute oral toxicity study was conducted as per the internationally accepted protocol of the OECD Guideline 425 [37]. Fifteen healthy female non-pregnant and nulliparous mice aged 6–8 weeks and weighing 22–28 g were randomly assigned to 3 groups, each having 5 mice. All mice were fasted (food only) for 4 h before and 2 h after administration of the test substances. The test substances were dissolved in 2% Tween 80 (2% TW80). One mouse from each group was orally administered 2000 mg/kg of RM-M (Group 1), RM-H (Group 2), and RM-H1 (Group 3), consecutively. Then the mice were observed for general signs and symptoms of toxicity and mortality within 24 h. The mouse receiving 2000 mg/kg of the RM-M (Group 1) survived, but the mouse from Group 2 and the mouse from Group 3 died 30 min after administration. Since no death was observed within 24 h, 2000 mg/kg of RM-M was administered to the remaining 4 mice in Group 1. Then they were observed individually for general signs and symptoms of toxicity; physical or behavioral changes, such as loss of appetite, ruffled fur, lacrimation, and mortality; and other signs of toxicity. The observation was carried out for 4 h with 30 min intervals and then for 14 consecutive days with an interval of 24 h [37].

The main test was conducted for acute oral toxicity study of RM-H and RM-H1. Since there was no information regarding the 50% lethal dose (LD_50_) and the slope of the dose-response curve for both test substances, dosing was initiated at 175 mg/kg [37]. Twelve mice were grouped into two groups of six mice each, and one mouse from each group received 175 mg/kg of the test substances (RM-H (Group 1) and RM-H1 (Group 2)). Since the experimental animals survived for 48 h, doses of the test substances were increased by a factor of 3.2 to 550 mg/kg. Following this, 550 mg/kg of RM-H and RM-H1 were given to a second mouse from Group 1 and Group 2, respectively, which resulted in the death of both mice 4 h after administration. Therefore, the remaining 4 mice in Group 1 received 175 mg/kg/mouse of RM-H while those in Group 2 were given 175 mg/kg/mouse of RM-H1. The mice were observed individually for general signs and symptoms of toxicity; physical or behavioral changes such as loss of appetite, ruffled fur, lacrimation, and mortality; and other signs of toxicity continuously for 4 h with 30 min intervals and then for 14 consecutive days with an interval of 24 h [37].

### 3.9. In Vivo Antimalarial Assay

#### 3.9.1. Inoculation of Mice

The parasitemia of the donor mice was determined by preparing blood smears on microscope slides from blood film taken from the tails of infected mice [38]. The smear was fixed with methanol and stained with Giemsa to determine the parasitemia level of the donor under a microscope. When the parasitemia level was 30–40%, parasitized erythrocytes were collected from the donor mouse by cardiac puncture using a sterile syringe and placed in a Petri dish containing an anticoagulant (0.5% trisodium citrate) and then immediately diluted with uninfected mouse blood and normal saline (0.9%) in such way that the final volume contained 5 × 10^7^ infected erythrocytes/mL of blood [39]. The diluted blood (0.2 mL) was then injected into all the experimental mice intraperitoneally (IP) [40].

#### 3.9.2. 4-Day Suppressive Test (Peter’s Test)

A four-day suppressive test with mice infected with chloroquine-sensitive *P. berghei* was employed according to the method described previously [41]. Fifty-five mice were injected with an inoculum of 1 × 10^7^ *P. berghei*-infected erythrocytes IP on the first day (day 0) [42]. Two h post-infection, the mice were randomly distributed into eleven groups, each containing five mice. Group 1 served as a negative control group (received vehicle 2% Tween 80, 10 mL/kg/day) and Group 2 as positive control group (received chloroquine, 25 mg/kg/day). The remaining nine groups were treatment groups. Groups 3–5 received 100, 200, and 400 mg/kg/day of RM-M, respectively, while Groups 6–8 and Groups 9–11 received 8.75, 17.50, and 35.00 mg/kg/day RM-H and RMH-1, respectively. All test substances were administered orally using oral gavage, and the doses were determined based on the acute oral toxicity test results. The middle dose was one tenth of the safe dose (~2000 mg/kg for RM-M and ~175 mg/kg for RM-H and RM-H1). The higher dose was twice the middle dose, and the lower dose was half of the middle dose [43,44]. Treatment was started 3 h post-infection on day 0 and continued for an additional three consecutive days at 24, 48, and 72 h post-infection (until day 3). On day 4 of the experiment (at 96 h post-infection), blood was collected from the tail of each mouse, and a thin smear was prepared on a microscope slide to determine parasitemia [45]. In addition, body weight, rectal temperature, and PCV were measured just before infection and at the end of the experiment [46]. Afterwards, mice were observed for 28 days (day 0–27) to determine the MST for each group [47].

#### 3.9.3. Rane’s Test

Rane’s test, which evaluates the curative potential of RM-H1, was performed using the method described earlier [48]. Twenty-five mice were injected IP with an inoculum of 1 × 10^7^ *P. berghei*-infected erythrocytes on the first day (Day 0) [49]. At day 3, the animals were randomly divided into five groups with five mice in each group. Group 1 served as the negative control group and received the vehicle (2% Tween 80, 10 mL/kg/day), and Group 2 served as the positive control group and received chloroquine at 25 mg/kg/day. Groups 3–5 were treated with 8.75, 17.50, and 35.00 mg/kg/day RM-H1, respectively. Treatment continued for a further 3 days (i.e., 96, 120, and 144 h post-infection) [50]. Parasitemia levels were recorded daily throughout the experiment starting at day 3 [51]. PCVs, rectal temperatures, and body weights were measured just before the first dose (day 3) and at the end of the experiment (day 7). Thereafter, all groups were observed for 28 days, and their survival times were recorded [52].

#### 3.9.4. Prophylactic Test

Investigation of the prophylactic potential of RM-H1 was done following the method described previously [53]. Twenty-five mice were randomly assigned into five groups of five mice each. Group 1, which served as negative control received vehicle (2% TW80, 10 mL/kg/day) and Group 2 (the positive control group) received chloroquine 25 mg/kg/day. Groups 3–5 were treated with 8.75, 17.50 and 35.00 mg/kg/day of RM-H1, respectively_._ Treatment was given orally for 3 days, 24 h after the last treatment (day 0), all mice were infected with an inoculum of 1 × 10^7^ *P. berghei*-infected blood [45,49]. Seventy-two h post-infection (day 3), blood smears were prepared from each mouse and the parasitemia level was determined [50]. PCV rectal temperature and body weight were measured just before parasite inoculation (day 0) and at the end of the experiment (day 3) [51]. Finally, the groups were followed for 28 days in order to record their survival time [52].

### 3.10. Determination of Parasitemia and Survival Time

Blood from each mouse was applied as a thin smear onto different microscope slides. Then the smear was fixed with methanol for 15 min and stained with 10% Giemsa for 15 min. The slides were washed with tap water and dried at room temperature. The number of parasite-infected RBCs were counted using a light microscope with an oil immersion objective lens at a magnification power of 100×. Parasitemia was determined by counting a minimum of three fields per slide. The percent of parasitemia and percent of inhibition were calculated by the following Peters–Robinson formula [54].

$$\%\;\mathrm{Parasitemia}\;=\;\frac{\mathrm{Number}\;\mathrm{of}\;\mathrm{parasitized}\;\mathrm{RBC}}{\mathrm{Total}\;\mathrm{number}\;\mathrm{of}\;\mathrm{RBC}\;\mathrm{count}}\;\times \;100$$

$$\%\;\mathrm{Suppression}\;=\frac{\%\;\mathrm{Parasitemia}\;\mathrm{in}\;\mathrm{negative}\;\mathrm{control}\;-\;\%\;\mathrm{Parasitemia}\;\mathrm{in}\;\mathrm{the}\;\mathrm{study}\;\mathrm{group}}{\%\;\mathrm{Parasitemia}\;\mathrm{in}\;\mathrm{negative}\;\mathrm{control}}\;\times \;100$$

The MST was determined using the formula indicated below:

$$\mathrm{Mean}\;\mathrm{survival}\;\mathrm{time}\;\left(\mathrm{MST}\right)\;=\;\frac{\mathrm{Sum}\;\mathrm{of}\;\mathrm{survival}\;\mathrm{time}\;\mathrm{of}\;\mathrm{all}\;\mathrm{mice}\;\mathrm{in}\;a\;\mathrm{group}\;\left(\mathrm{daily}\right)}{\mathrm{Total}\;\mathrm{number}\;\mathrm{of}\;\mathrm{mice}\;\mathrm{in}\;\mathrm{the}\;\mathrm{group}}$$

### 3.11. Determination of Packed Cell Volume, Rectal Temperature, and Body Weight

Blood was collected from the tail of each mouse in heparinized microhematocrit capillary tubes, filling up to three-fourths of the tube, and then the tube was sealed at the dry end with sealing clay. The tubes were then placed in a microhematocrit centrifuge with the sealed ends out wards. The blood was centrifuged at 12,000 rpm for 10 min. The PCV was determined using the following formula.

$$\mathrm{PCV}\;=\;\frac{\mathrm{Volume}\;\mathrm{of}\;\mathrm{erythrocytes}\;\mathrm{in}\;a\;\mathrm{given}\;\mathrm{volume}\;\mathrm{of}\;\mathrm{blood}}{\mathrm{Total}\;\mathrm{blood}\;\mathrm{volume}}\;\times \;100$$

All mice were weighed using a sensitive digital weighing balance, and a digital rectal thermometer was used to measure rectal temperature. The percentage changes of mean values of all three parameters that occurred before and after treatment were then calculated.

$$\%\;\mathrm{Change}\;=\;\frac{\mathrm{Mean}\;\mathrm{on}\;\mathrm{final}\;\mathrm{day}\;-\;\mathrm{Mean}\;\mathrm{on}\;\mathrm{initial}\;\mathrm{day}}{\mathrm{Mean}\;\mathrm{on}\;\mathrm{final}\;\mathrm{day}}\;\times \;100$$

### 3.12. In Vitro Antimycobacterial Assay

The MICs were determined against *M. smegmatis* mc^2^ 155 pTEC27 and *M. abscessus* ATCC 19977 pTEC27 by the broth microdilution method with 96-Well flat-bottom tissue culture plates (Sarstedt, 83.3924.500) [35]. In the third well of each row, two times of the desired highest concentration of the tested compound was added in a 7H9 medium supplemented with 10% ADS and 0.05% polysorbate 80. Each compound was diluted twofold in a ten-point serial dilution. The concentration of the starting inoculum was 5 × 10^5^ cells mL^−1^. The starting inoculum was diluted from a preculture at the mid-log phase (OD_600_ 0.3 to 0.7), and an OD_600_ of 0.1 was correlated to 1 × 10^8^ CFU mL^−1^. The plates were sealed with parafilm, placed in a container with moist tissue, and incubated for three days at 37 °C. Each plate had eight negative controls [1% dimethyl sulfoxide (DMSO)] and eight positive controls (100 µM amikacin). After incubation, the plates were monitored by OD measurement at 590 nm (Tecan SpectraFluor). The assay was performed in duplicate, and the results were validated by RFP measurement.

Every assay plate contained eight wells with 1% DMSO as a negative control, which corresponds to 100% bacterial growth, and eight wells with amikacin (100 µM) as a positive control, in which 100% inhibition of bacterial growth was reached. The controls were used to monitor the assay quality through determination of the z-score. The z-factor was calculated as follows:

$$Z\prime \;=\;1\;-\;\frac{3\;({\mathrm{SD}}_{\mathrm{amikacin}}\;+\;{\mathrm{SD}}_{\mathrm{DMSO}})}{M_{\mathrm{amikacin}}\;-\;M_{\mathrm{DMSO}}}$$

where SD stands for standard deviation and M for mean.

The percentage of growth inhibition was calculated by the equation:

$$\%\;\mathrm{Growth}\;\mathrm{inhibition}\;=\;-100\%\;\times \;\frac{{\mathrm{OD}}_{590}\left(\mathrm{sample}\right)\;-\;{\mathrm{OD}}_{590}\left(\mathrm{DMSO}\right)}{{\mathrm{OD}}_{590}\left(\mathrm{DMSO}\right)-{\mathrm{OD}}_{590}\left(\mathrm{amikacin}\right)}$$

where OD represents optical density.

### 3.13. Statistical Analysis

The data analysis was carried out using IBM SPSS (Statistical Package for Social Sciences) Statistics for Windows, Version 25.0. The results were expressed as mean ± standard error of mean (M ± SEM). Statistical significance was determined by a one-way ANOVA followed by the Tukey post hoc test to compare different parameters among the treatment and control groups. A result of *p* < 0.05 was considered significant.

## 4. Conclusions

In the present study, the initial bioassay tests conducted on the 80% methanol and hydrodistilled leaf extracts of *R. multifidus* indicated the antimalarial potency of the plant. The hydrodistilled extract was proven to express the highest potency in inhibiting growth of this pathogenic protozoan parasite. Further phytochemical analysis of the hydrodistilled extract resulted in the isolation of an α,β-unsaturated dilactone characterized as anemonin. The investigation for antiprotozoal activity has demonstrated that anemonin exerts a strong potency for inhibiting the growth of the parasite, suggesting that it is responsible, in full or in part, for the antimalarial activity of the plant. To our knowledge, this study is the first to demonstrate the antimalarial activity of anemonin that has been isolated from the leaves of *R. multifidus*. Finally, the results produced in this study may serve as an additional reference in natural product research and may contribute to the further study of antiprotozoal drug discovery. The findings also support the use of the plant in the treatment of malaria in traditional medicine. The lack of toxicity and some specificity of the anti-infective effect, which is shown by a lack of growth inhibition of anemonin against mycobacteria, add to the safety and effectivity of the plant extract and its major active constituent. However, the present work has limitations in that the mode of action of the extract and anemonin against the parasite was not studied. Thus, further study to confirm the capability of anemonin to interact with the thiol group of cysteine by a Michael-type addition reaction needs to be carried out. Moreover, the GSH levels in the parasitized blood of mice should be measured in vitro in the presence and absence of anemonin to gain some insight into the mechanism(s) of action of the plant extract.

## Figures and Tables

**Table 1:** Antimalarial activity of the leaf extracts of *Ranunculus multifidus* in mice infected with *Plasmodium berghei* in 4-day suppressive test.

Test Substances (mg/kg/day)	% Parasitemia	% Suppression	Mean Survival Time (in Days)
2% TW80	26.6 ± 0.7	-	9.1 ± 0.3
CQ 25	0.2 ± 0.2 ^a3^	99.2	28 ± 0.0 ^a2^
RM-M 100	15.8 ± 1.0 ^a2,b2,c2,d1,e2,f2,g2^	39.2	12.4 ± 0.4 ^a2,b2,g1^
RM-M 200	12.6 ± 0.2 ^a2,b2,e2,f2,g2^	48.4	12.8 ± 0.5 ^a2,b2,g1^
RM-M 400	9.5 ± 0.2 ^a2,b2,g1,g2^	53.1	13.1 ± 0.2 ^a2,b2^
RM-H 8.75	10.9 ± 0.5 ^a2,b2,g2^	58.8	13.8 ± 0.8 ^a2,b2^
RM-H 17.50	9.6 ± 0.4 ^a2,b2,g1^	63.6	14.0 ± 0.8 ^a2,b2^
RM-H 35.00	15.8 ± 1.0 ^a2,b2,^	70.6	14.4 ± 0.5 ^a2,b2^

Data are expressed as mean ± SEM; *n* = 5; a: compared to 2% TW80 (Tween 80) vehicle, b: compared to chloroquine 25, c: compared to RM-M 200, d: compared to RM-M 400, e: compared to RM-H 8.75, f: compared to RM-H 17.50, g: compared to RM-H 35.00; 1: *p*  <  0.05, 2: *p* < 0.001; CQ: chloroquine phosphate, RM-M: 80% methanol extract of *R. multifidus*, RM-H: hydrodistilled extract of *R. multifidus*.

**Table 2:** Effect of leaf extracts of *Ranunculus multifidus* on body weight, rectal temperature and packed cell volume of *Plasmodium berghei*-infected mice in 4-day suppressive test.

Test Substances (mg/kg/day)	Body Weight (g)			Rectal Temperature (°C)			Packed Cell Volume (%)		
	Day 0	Day 4	% Change	Day 0	Day 4	% Change	Day 0	Day 4	% Change
2% TW80	26.0 ± 0.2	22.1 ± 0.6	−14.7	36.5 ± 0.0	33.0 ± 0.2	−9.6	59.2 ± 0.2	48.6 ± 0.9	−17.9
CQ 25	26.3 ± 0.5	29.2 ± 0.6	11.3 ^a3^	36.6 ± 0.1	36.5 ± 0.1	−0.0 ^a3^	57.2 ± 0.5	58.8 ± 0.7	2.7 ^a3^
RM-M 100	26.6 ± 0.9	26.8 ± 0.9	0.7 ^a3,b3,c1,d3,e3,f3,g3^	36.4 ± 0.2	35.1 ± 0.2	−3.6 ^a3,b3,d1,e1,f3,g3^	56.4 ± 0.7	50.2 ± 0.5	−10.9 ^a1,b3^
RM-M 200	26.8 ± 0.9	27.9 ± 1.0	3.9 ^a3,b3,d3,e3,f3,g3^	36.4 ± 0.1	35.5 ± 0.1	−2.3 ^a3,b3,d1,g2^	58.0 ± 0.7	52.2 ± 0.8	−10.0 ^a3,b3^
RM-M 400	26.5 ± 1.0	28.3 ± 1.1	6.6 ^a3,b3,e1,f3,g3^	36.6 ± 0.0	36.2 ± 0.2	−1.0 ^a3,b1,e1,f1,g3^	57.4 ± 0.9	52.2 ± 0.5	−9.0 ^a3,b3^
RM-H 8.75	25.3 ± 0.9	26.6 ± 0.9	5.0 ^a3,b3,e1,g3^	36.2 ± 0.0	35.1 ± 0.2	−2.0 ^a3,b3,g2^	57.2 ± 0.8	51.2 ± 0.7	−10.4 ^a3,b3^
RM-H 17.50	24.8 ± 0.5	26.8 ± 0.9	7.8 ^a3,b1,g2^	36.5 ± 0.1	35.8 ± 0.1	−1.9 ^a3,b3,g2^	55.6 ± 0.5	50.0 ± 0.5	−10.0 ^a3,b3^
RM-H 35.00	26.1 ± 0.6	28.7 ± 1.0	10.0 ^a3^	36.6 ± 0.1	36.4 ± 0.1	−0.7 ^a3^	57.6 ± 0.5	52.4 ± 0.6	−9.0 ^a3,b3^

Data are expressed as mean ± SEM; *n* = 5; a: compared to 2% TW80 (Tween 80) vehicle, b: compared to chloroquine, c: compared to RM-M 200, d: compared to RM-M 400, e: compared to RM-H 8.75, f: compared to RM-H 17.50, g: compared to RM-H 35.00; 1: *p* < 0.05, 2: *p* < 0.01, 3: *p* < 0.001; CQ: chloroquine phosphate, RM-M: 80% methanol extract of *R. multifidus*, RM-H: hydrodistilled extract of *R. multifidus*.

**Table 3:** Comparison of the ^1^H-NMR and ^13^C-NMR spectral data of the RM-H1 with ^1^H-NMR and ^13^C-NMR of anemonin reported earlier [17].

No.	^1^H NMR Chemical Shift (δ, ppm)		^13^C NMR Chemical Shift (δ, ppm)	
	Anemonin [17]		Anemonin [17]	
2 − 2′	-	-	171.8	170.8
3 − 3′	6.32, d (5.7Hz)	6.30, d (5.6Hz)	121.1	121.1
4 − 4′	8.28, d (5.7Hz)	8.26, d (5.6Hz)	156.5	153.2
5 − 5′	-	-	90.44	90.3
6 − 6′	6a − 6a′ = 2.64 6b − 6b′ = 2.25	6a − 6a′ = 2.61, m	23.27	23.8
		6b − 6b′ = 2.24, m		

d = doublet, m = multiplet.

**Table 4:** Antimalarial activity of anemonin in mice infected with *Plasmodium berghei* in 4-day suppressive test.

Test Substance (mg/kg/day)	% Parasitemia	% Suppression	Mean Survival Time (in Days)
2% TW80	26.6 ± 0.8	-	9.2 ± 0.4
Chloroquine	0.1 ± 0.3 ^a1^	99.5	28.0 ± 0.0 ^a1^
Anemonin 8.75	5.7 ± 0.1 ^a1,b1^	78.3	14.0 ± 0.3 ^a1,b1^
Anemonin 17.50	5.2 ± 0.1 ^a1,b1^	80.1	14.4 ± 0.2 ^a1,b1^
Anemonin 35.00	4.5 ± 0.0 ^a1,b1^	83.0	14.8 ± 0.4 ^a1,b1^

Data are expressed as mean ± SEM; *n* = 5; a: compared to 2% TW80 (Tween 80) vehicle, b: compared to CQ, 1: *p* < 0.001; CQ: chloroquine phosphate.

**Table 5:** Effect of anemonin on body weight, rectal temperature, and packed cell volume of mice infected with *Plasmodium berghei* in four-day suppressive antimalarial test.

Test Substances (mg/kg/day)	Body Weight (g)			Rectal Temperature (℃)			Packed Cell Volume (%)		
	Day 0	Day 4	% Change	Day 0	Day 4	% Change	Day 0	Day 4	% Change
2% TW80	25.0 ± 0.4	21.2 ± 0.5	−15.1	36.6 ± 0.0	33.0 ± 0.2	−9.9	59.6 ± 0.2	49.2 ± 0.9	−17.4
CQ 25	26.2 ± 0.5	30.5 ± 0.8	16.4 ^a3^	36.4 ± 0.3	36.4 ± 0.1	0.1 ^a3^	58.4 ± 0.3	58.8 ± 0.5	0.6 ^a3^
Anemonin 8.75	24.8 ± 0.0	26.8 ± 0.9	7.8 ^a3^	36.5 ± 0.1	35.8 ± 0.1	−1.6 ^a3^	57.2 ± 0.8	51.2 ± 0.7	−10.4 ^a3,b2^
Anemonin 17.50	26.1 ± 0.6	28.7 ± 1.0	10.0 ^a3^	36.7 ± 0.1	36.4 ± 0.1	−0.7 ^a3^	56.8 ± 0.8	52.4 ± 0.6	−7.7 ^a3,b3^
Anemonin 35.00	26.8 ± 0.9	30.3 ± 0.6	13.0 ^a3^	36.4 ±0.1	36.4 ± 0.1	0.0 ^a3^	56.4 ± 0.8	53.6 ± 0.5	−4.9 ^a3,b1^

Data are expressed as mean ± SEM; *n* = 5; a: compared to 2% TW80 (Tween 80) vehicle, b: compared to CQ; 1: *p* < 0.05, 2: *p* < 0.01, 3: *p* < 0.001; CQ: chloroquine phosphate.

**Table 6:** Antimalarial activity of anemonin in *Plasmodium berghei*-infected mice in Rane’s test.

Test Substances (mg/kg/day)	% Parasitemia	% Suppression	Mean survival Time (in Days)
2% TW80	78.0 ± 0.2	-	8.6 ± 0.1
CQ 25	1.1 ± 0.2 ^a1^	98.4	28.0 ± 0.0 ^a1^
Anemonin 8.75	47.2 ± 0.4 ^a1,b1^	38.8	11.6 ± 0.2 ^a1,b1^
Anemonin 17.50	45.9 ± 0.8 ^a1,b1^	40.5	12.0 ± 0.3 ^a1,b1^
Anemonin 35.00	44.3 ± 0.7 ^a1,b1^	42.6	13.0 ± 0.3 ^a1,b1^

Data are expressed as mean ± SEM; *n* = 5; a: compared to 2% TW80 (Tween 80) vehicle, b: compared to CQ; 1: *p* < 0.001; CQ: chloroquine phosphate.

**Table 7:** Body weight, rectal temperature, and packed cell volume of *Plasmodium berghei*-infected mice treated with anemonin in Rane’s antimalarial test.

Test Substances (mg/kg/day)	Body Weight (g)			Rectal Temperature (℃)			Packed Cell Volume (%)		
	Day 3	Day 7	% Change	Day 3	Day 7	% Change	Day 3	Day 7	% Change
2% TW80	26.6 ± 0.4	20.5 ± 0.8	−22.8	36.6 ± 0.2	32.2 ± 0.3	−11.8	58.2 ± 0.3	46.2 ± 0.4	−20.6
CQ 25	26.3 ± 0.7	27.3 ± 0.7	3.8 ^a1^	36.8 ± 0.0	36.3 ± 0.1	−1.3 ^a1^	58.0 ± 0.7	53.0 ± 0.7	−8.6 ^a1^
Anemonin 8.75	26.4 ± 0.8	23.6 ± 0.7	−10.7 ^b1^	36.7 ± 0.1	35.6 ± 0.2	−2.7 ^a1^	57.8 ± 0.5	51.4 ± 0.7	−10.6 ^a1^
Anemonin 17.50	28.5 ± 0.3	27.2 ± 1.1	−3.9 ^a1,b1^	36.7 ± 0.1	36.2 ± 0.0	−1.3 ^a1^	56.0 ± 0.7	50.2 ± 0.8	−10.3 ^a1^
Anemonin 35.00	26.3 ± 0.7	25.3 ± 0.7	−3.8 ^a1, b1^	36.8 ± 0.0	36.3 ± 0.3	−1.3 ^a1^	58.1 ± 0.7	53.0 ± 0.7	−8.7 ^a1^

Data are expressed as mean ± SEM; *n* = 5; a: compared to 2% TW80 (Tween 80) vehicle, b: compared to CQ; 1: *p* < 0.001; CQ: chloroquine phosphate.

**Table 8:** Prophylactic antimalarial activity of anemonin in *Plasmodium berghei*-infected mice.

Test Substances (mg/kg/day)	% Parasitemia	% Suppression	Mean Survival Time (in Days)
2% TW80	33.8 ± 0.9	-	8.4 ± 0.5
CQ 25	3.8 ± 0.7 ^a1^	88.6	27.4 ± 0.4 ^a1^
Anemonin 8.75	13.1 ± 0.4 ^a1,b1^	60.7	14.0 ± 0.3 ^a1,b1^
Anemonin 17.50	12.1 ± 0.5 ^a1,b1^	63.7	14.0 ± 0.3 ^a1,b1^
Anemonin 35.00	11.1 ± 0.3 ^a1,b1^	66.7	14.4 ± 0.2 ^a1,b1^

Data are expressed as mean ± SEM; *n* = 5; a: compared to 2% TW80 (Tween 80) vehicle, b: compared to CQ; 1: *p* < 0.001; CQ: chloroquine phosphate.

**Table 9:** Body weight, rectal temperature, and packed cell volume of *Plasmodium berghei*-infected mice treated with anemonin in prophylactic antimalarial test.

Test Substances (mg/kg/day)	Body Weight (g)			Rectal Temperature (℃)			Packed Cell Volume (%)		
	Day 3	Day 7	% Change	Day 3	Day 7	% Change	Day 3	Day 7	% Change
2% TW80	26.7 ± 0.5	21.6 ± 0.0	−19.0	36.6 ± 0.0	32.5 ± 0.2	−11.2	58.9 ± 0.4	47.8± 0.6	−18.8
CQ 25	26.1 ± 0.6	27.9 ± 0.5	6.8 ^a2^	36.7 ± 0.1	36.5 ± 0.1	−0.3 ^a2^	57.8 ± 0.5	53.4 ± 0.8	−7.6 ^a2^
Anemonin 8.75	26.4 ± 0.8	23.6 ± 0.7	−10.7 ^a2,b2^	36.7 ± 0.1	35.6 ± 0.2	−2.7 ^a2^	58.4 ± 0.6	51.0 ± 0.4	−12.6 ^a2,b1^
Anemonin 17.50	27.4 ± 0.5	27.5 ± 0.4	0.3 ^a2,b2^	36.5 ± 0.1	36.1 ± 0.2	−1.0 ^a2^	58.0 ± 0.5	50.8 ± 0.3	−12.4 ^a2,b1^
Anemonin 35.00	26.9 ± 0.3	27.2 ± 0.5	1.3 ^a2,b2^	36.5 ± 0.2	36.2 ± 0.0	−0.7 ^a2^	58.6 ± 0.5	53.4 ± 0.8	−8.8 ^a2^

Data are expressed as mean ± SEM; *n* = 5; a: compared to 2% TW80 (Tween 80) vehicle, b: compared to CQ; 1: *p* < 0.05, 2: *p* < 0.001; CQ: chloroquine phosphate.

## Supplementary Materials

The following are available online, TLC chromatograms, NMR, and HR-MS spectra of anemonin. Microscope slide photos of the negative control group (A), anemonin (B), and positive control group (C).

## Sample Availability

Samples of the compound are available from the authors.
