# Supplementary material for: In Vivo Antimalarial Activity of Leaf Extracts and a Major Compound Isolated from Ranunculus multifidus Forsk
Source: Molecules. 2021 Oct 13;26(20):6179. doi: 10.3390/molecules26206179 (PMC8537993; doi:10.3390/molecules26206179)
Supplement: Supplementary file 1 [file molecules-26-06179-s001.zip › molecules-1369146-supplementary.pdf]

Supplementary data

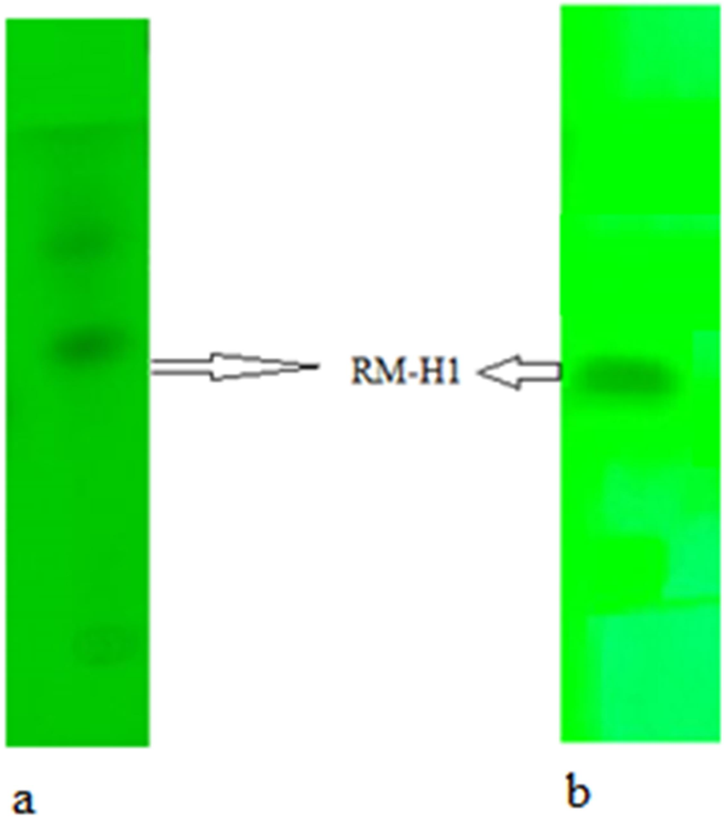

Silica gel TLC chromatograms of (a) the hydrodistilled extract (RM-H) of *Ranunculus multifidus* leaves in hexane: ethyl acetate (5:1) solvent system (b) purified RM-H1 in hexane: ethyl acetate (2:3) solvent system viewed under UV light of 254 nm

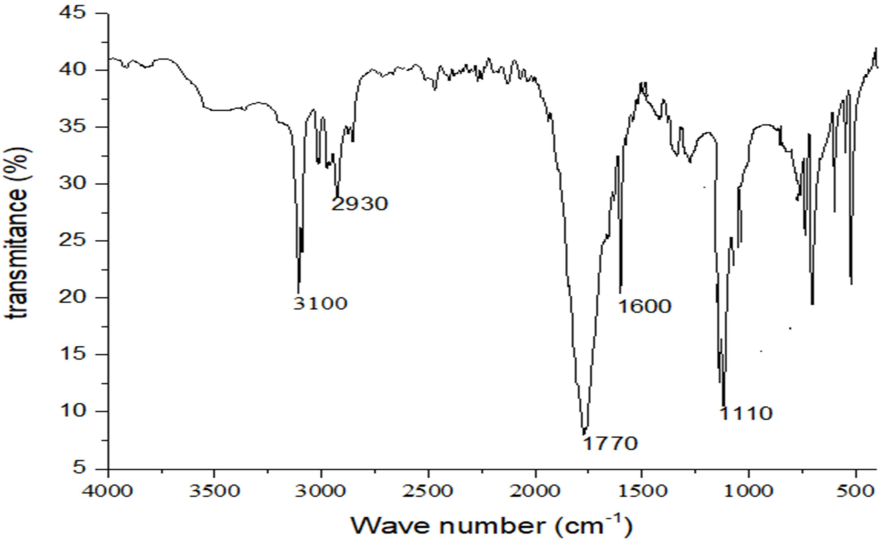

Fourier-transform infrared spectrum of anemonin

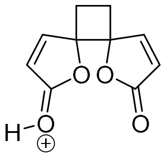

Chemical Formula: $C_{10}H_9O_4^+$
Exact Mass: 193,0495

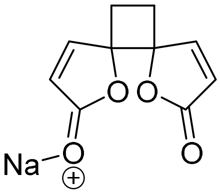

Chemical Formula: $C_{10}H_8NaO_4^+$
Exact Mass: 215,0315

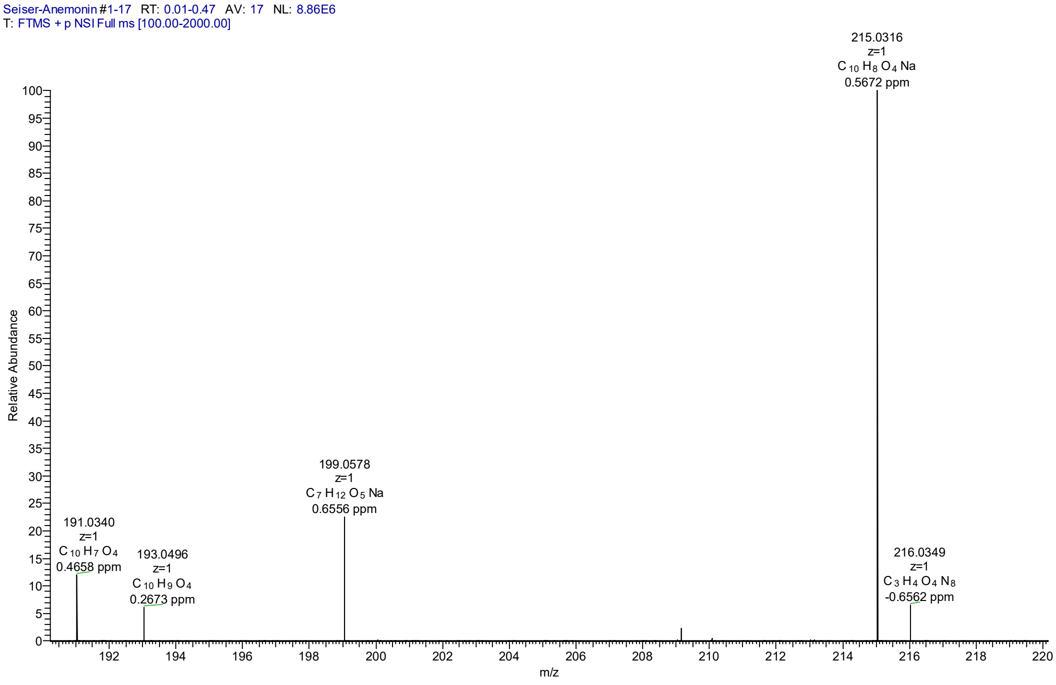

HRMS of anemonin

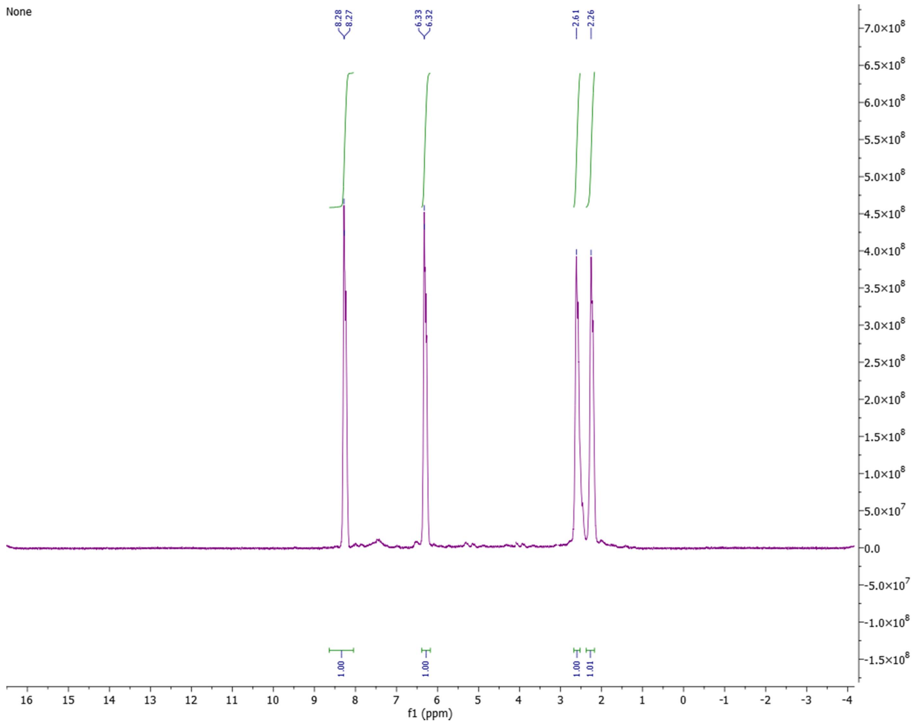

^{1}H-NMR spectrum of anemonin

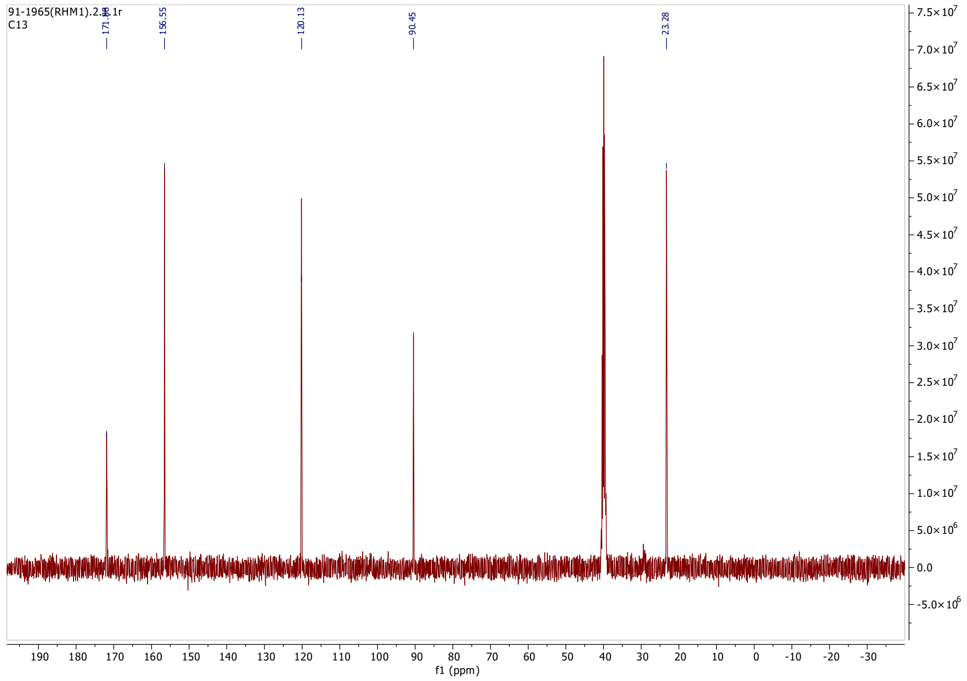

^{13}C-NMR spectrum of RM-H1

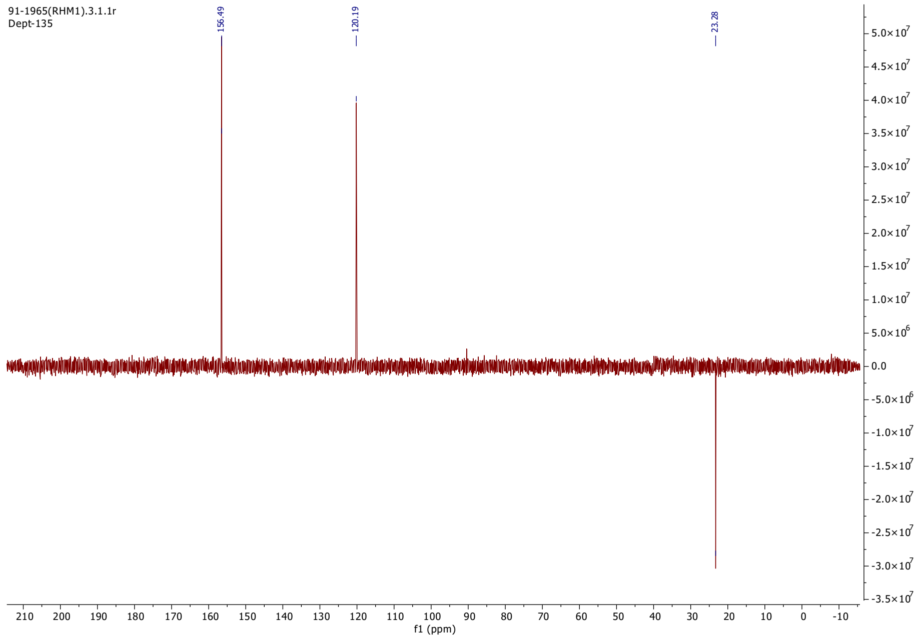

DEPT-135 spectrum of RM-H1

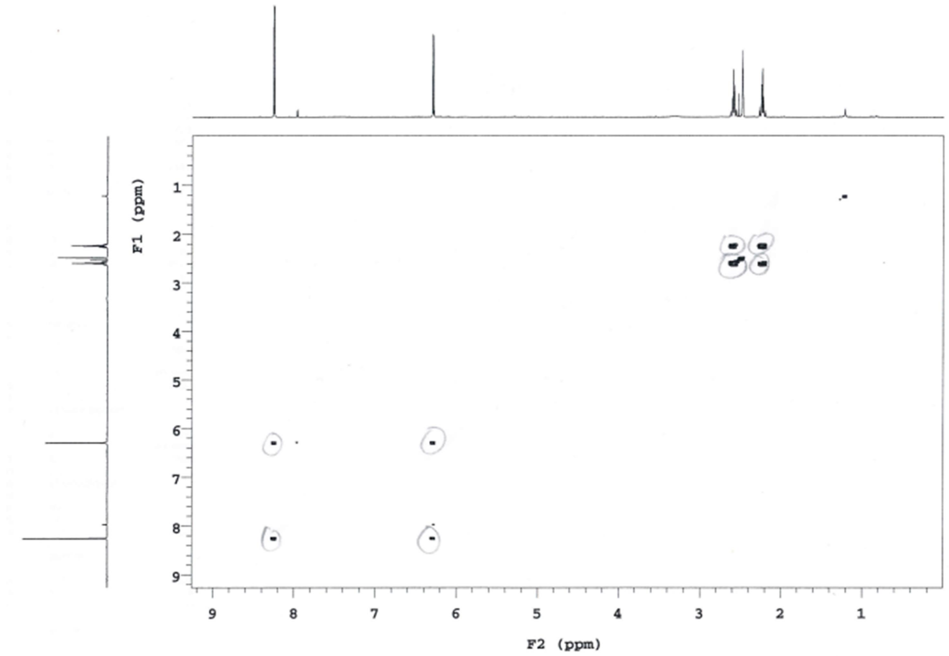

2D NMR ^{1}H-^{1}H COSY spectrum of anemonin

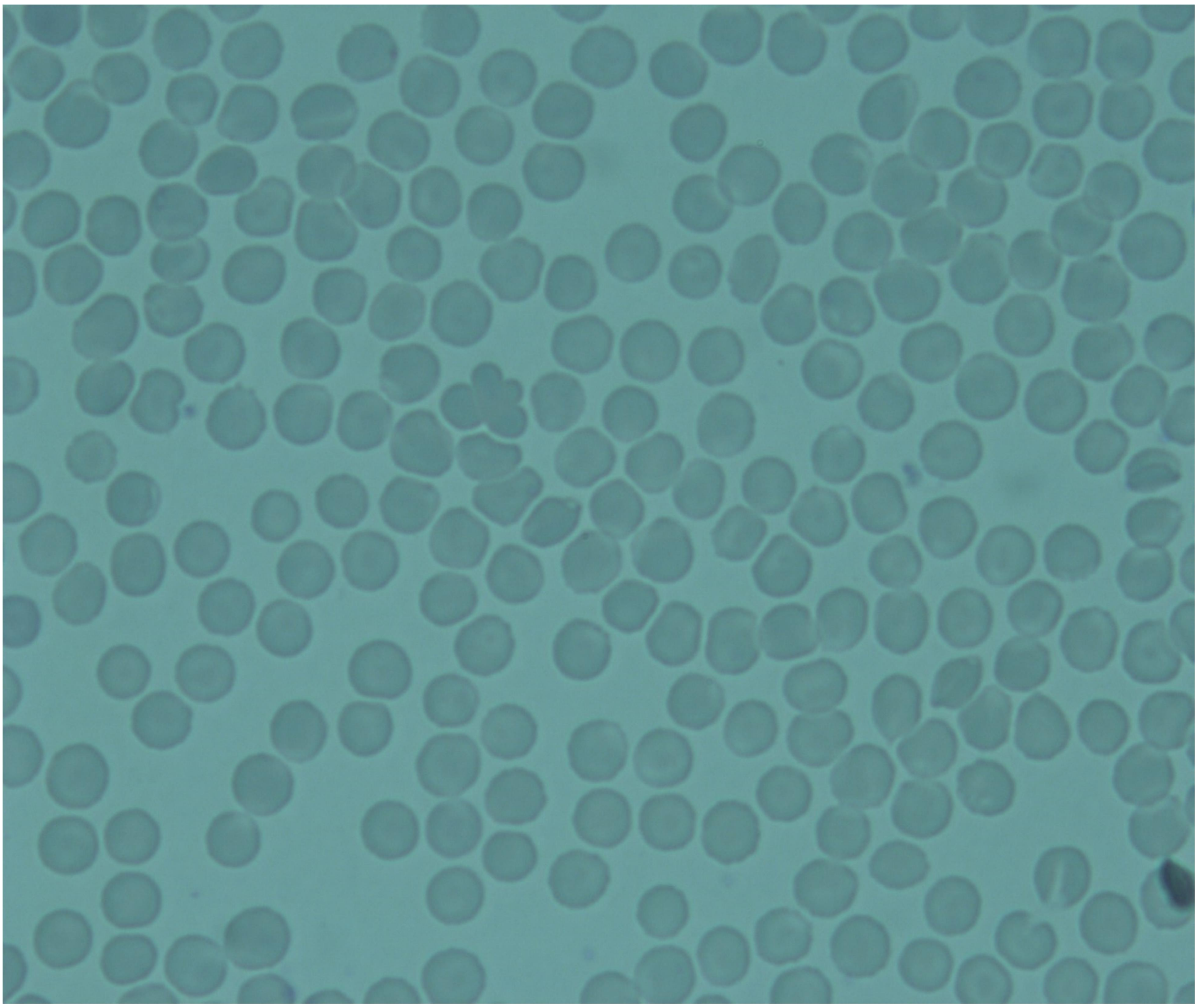

0.1% parasitaemia - chloroquine in 4 day

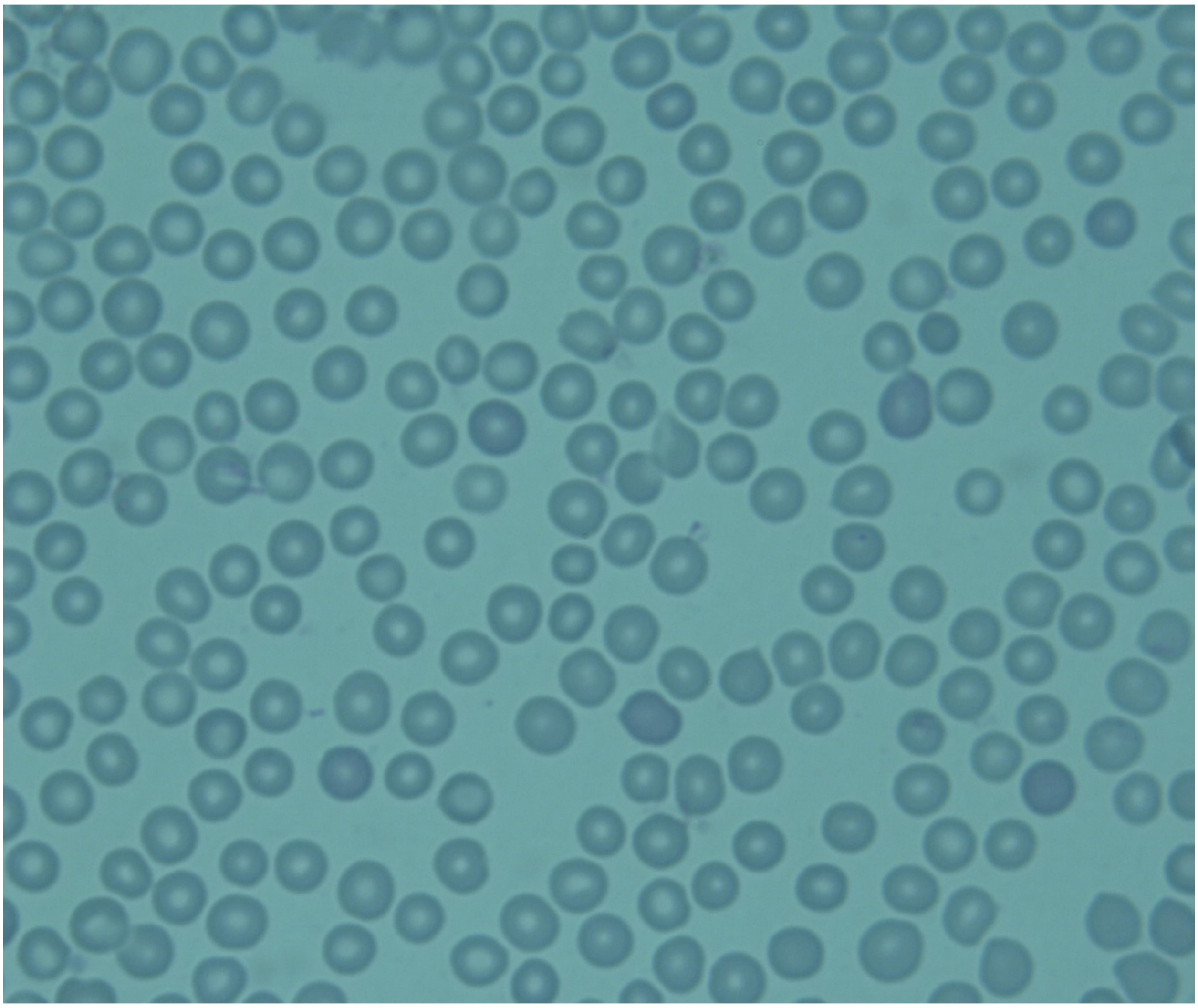

1.1% parasitaemia - chloroquine in Ranes test

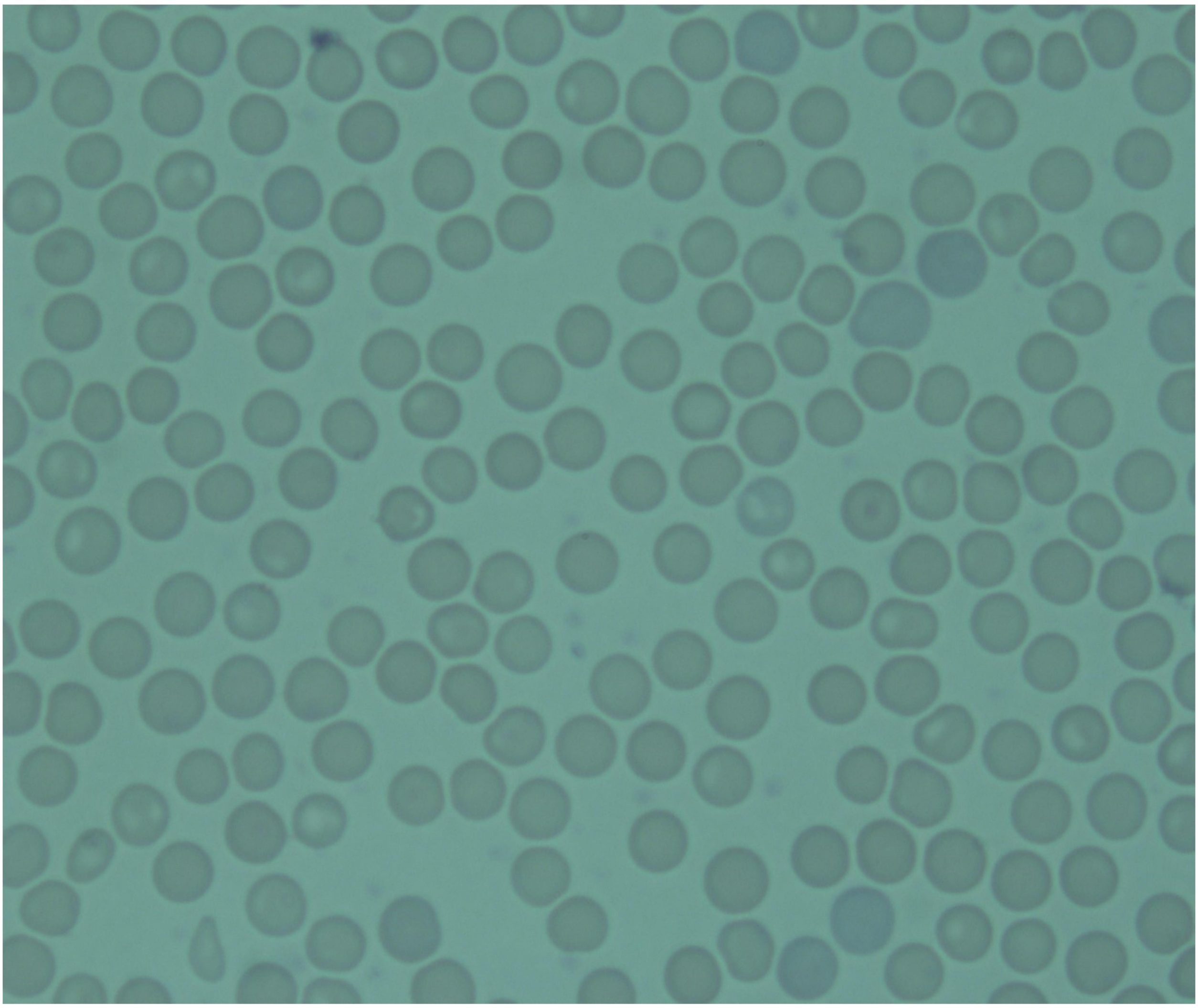

3.8 % parasitaemia - chloroquine in prophylactic

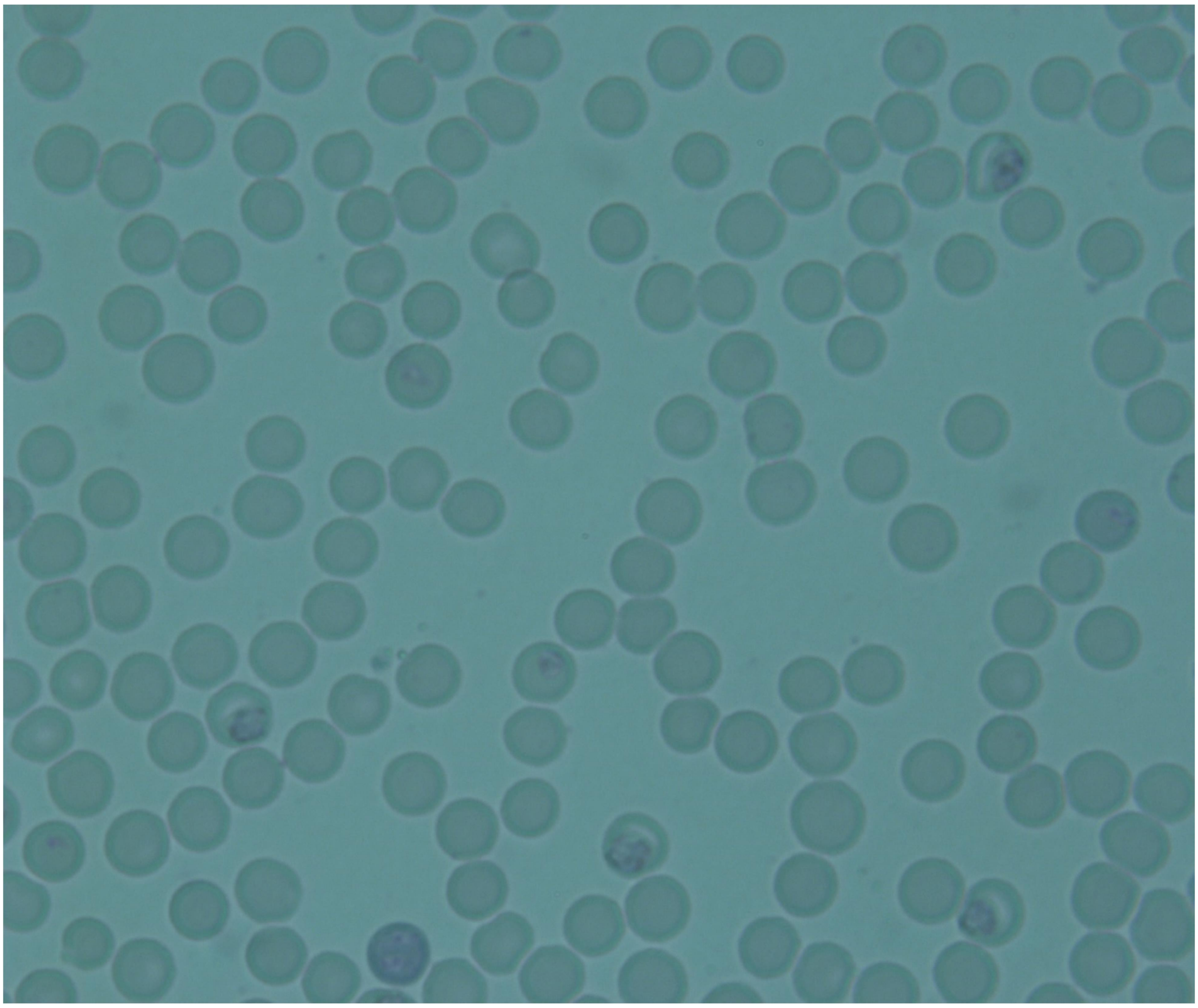

5% parasitaemia - anemonin in 4-day suppressive test

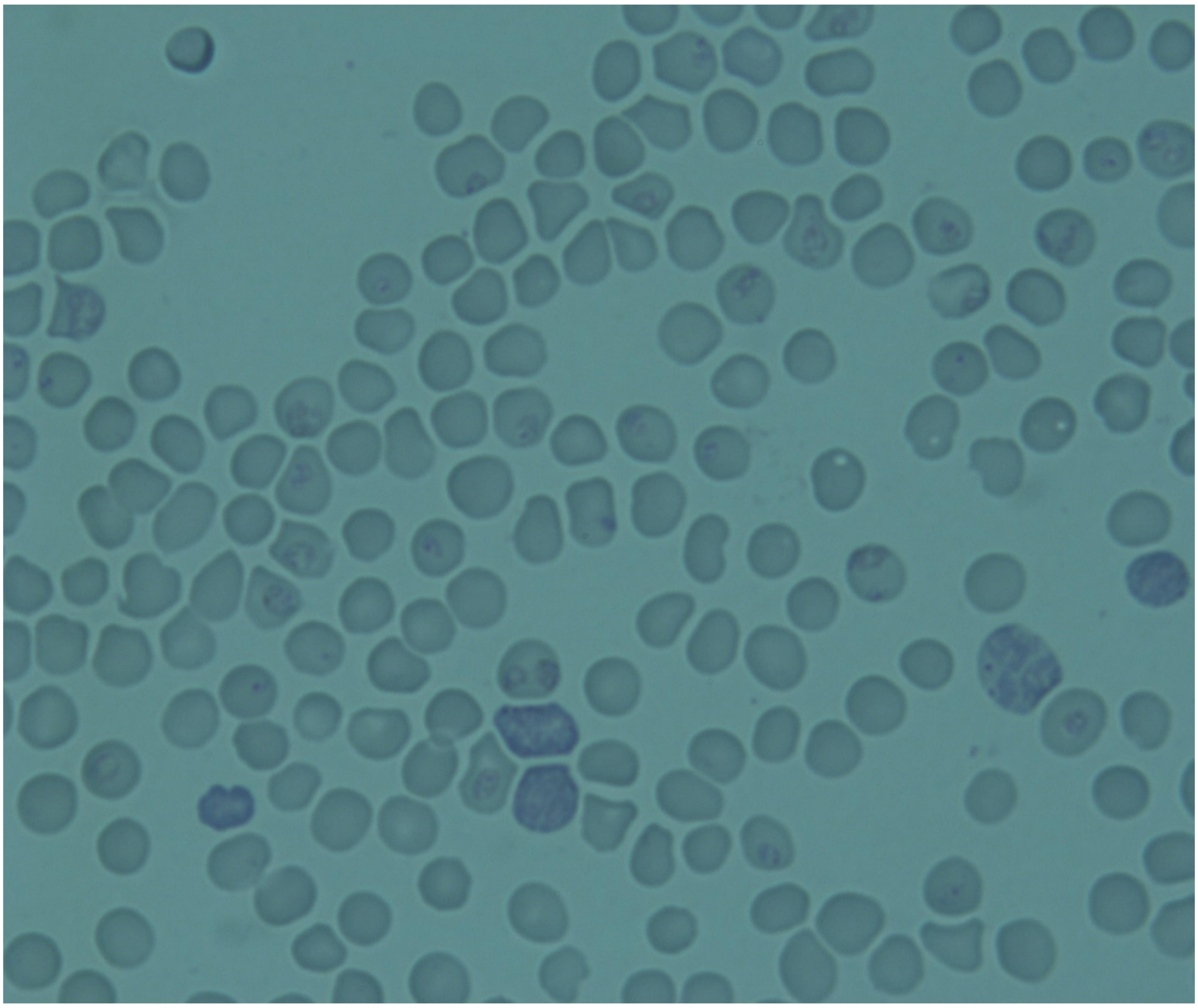

11% parasitaemia anemonin in prophylactic

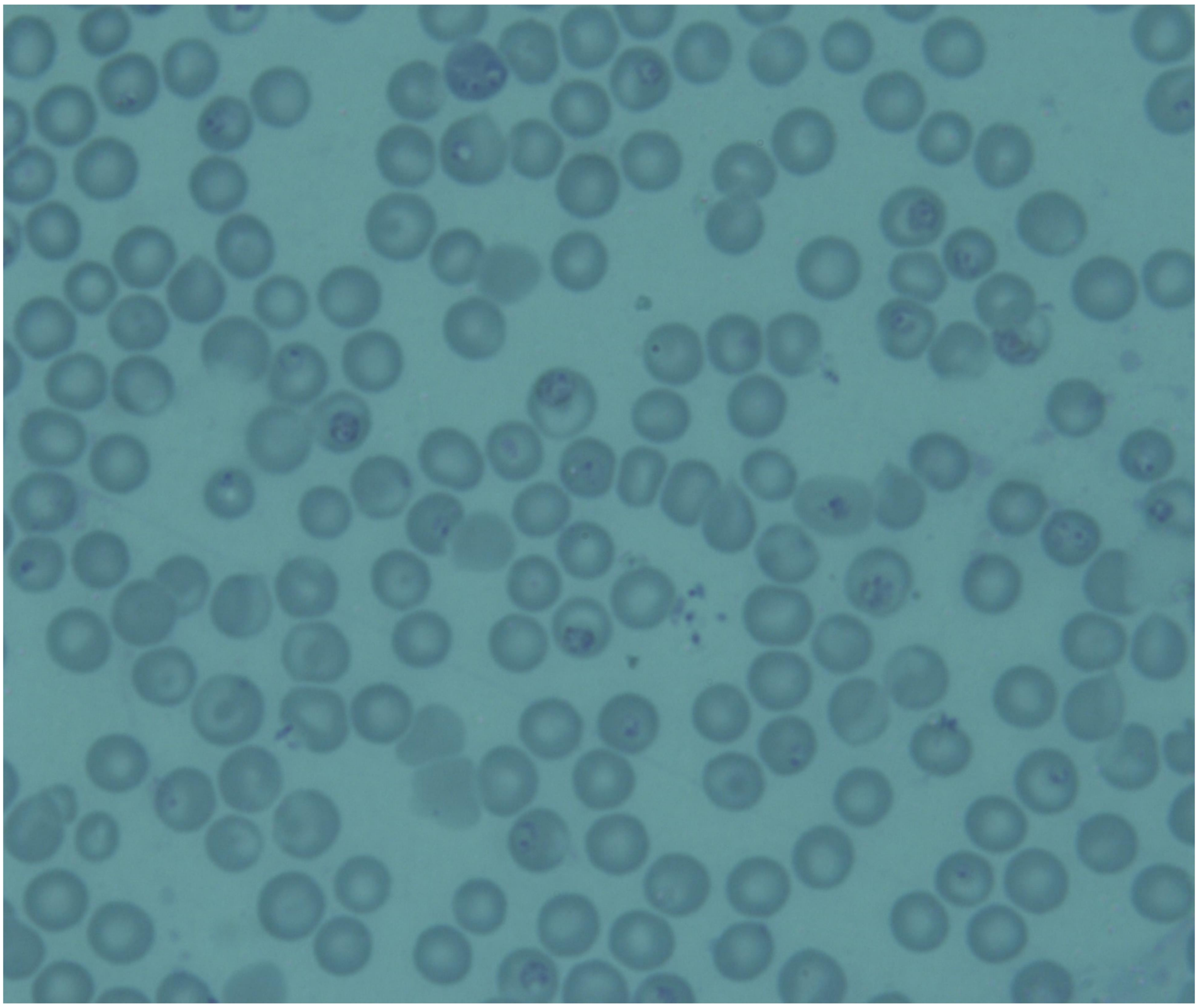

26.6% parasitaemia negative control in 4-day suppressive test 2% TW80

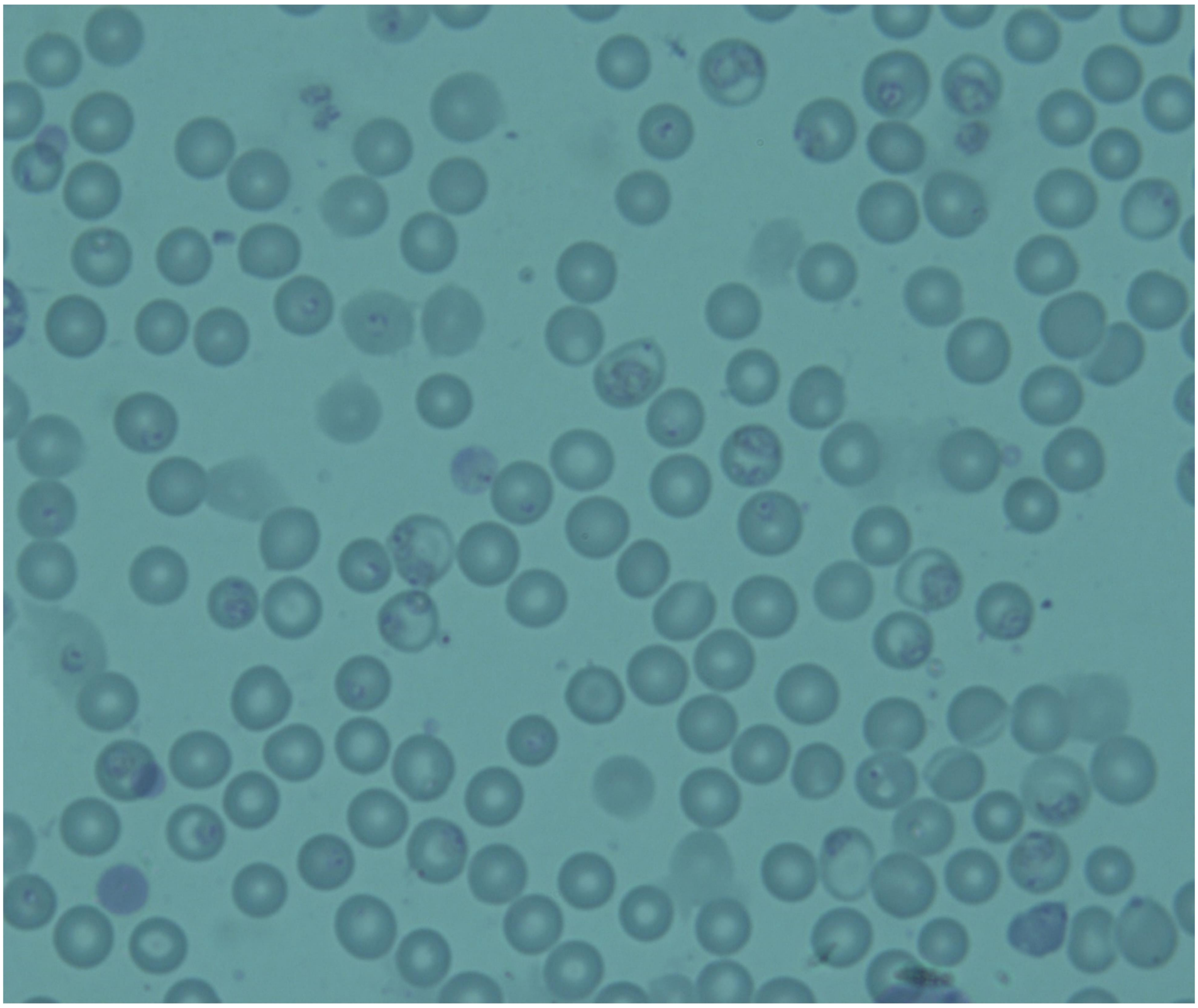

33% parasitaemia negative control in prophylactic test 2% TW80

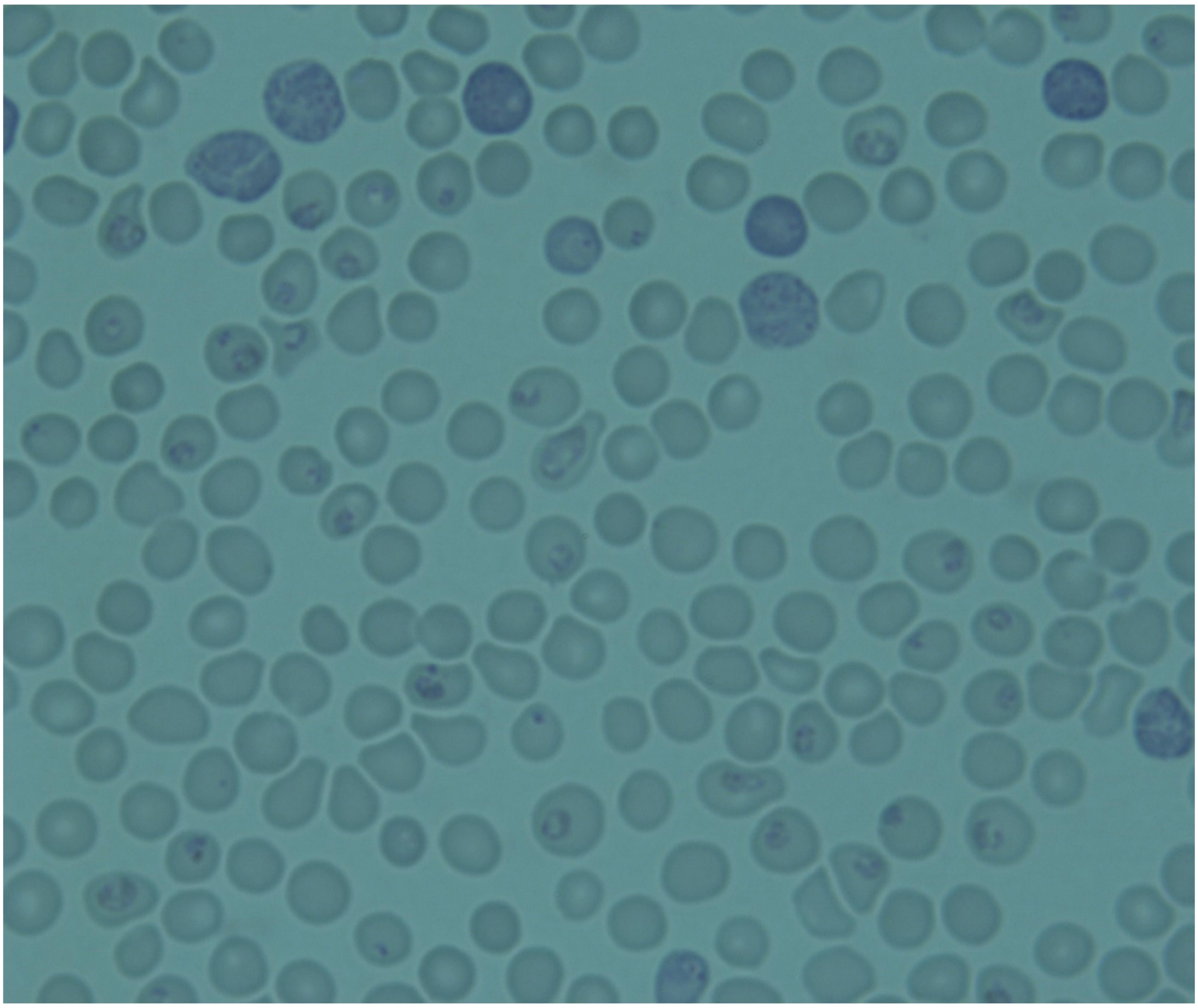

44% parasitaemia anemonin in Rane's test

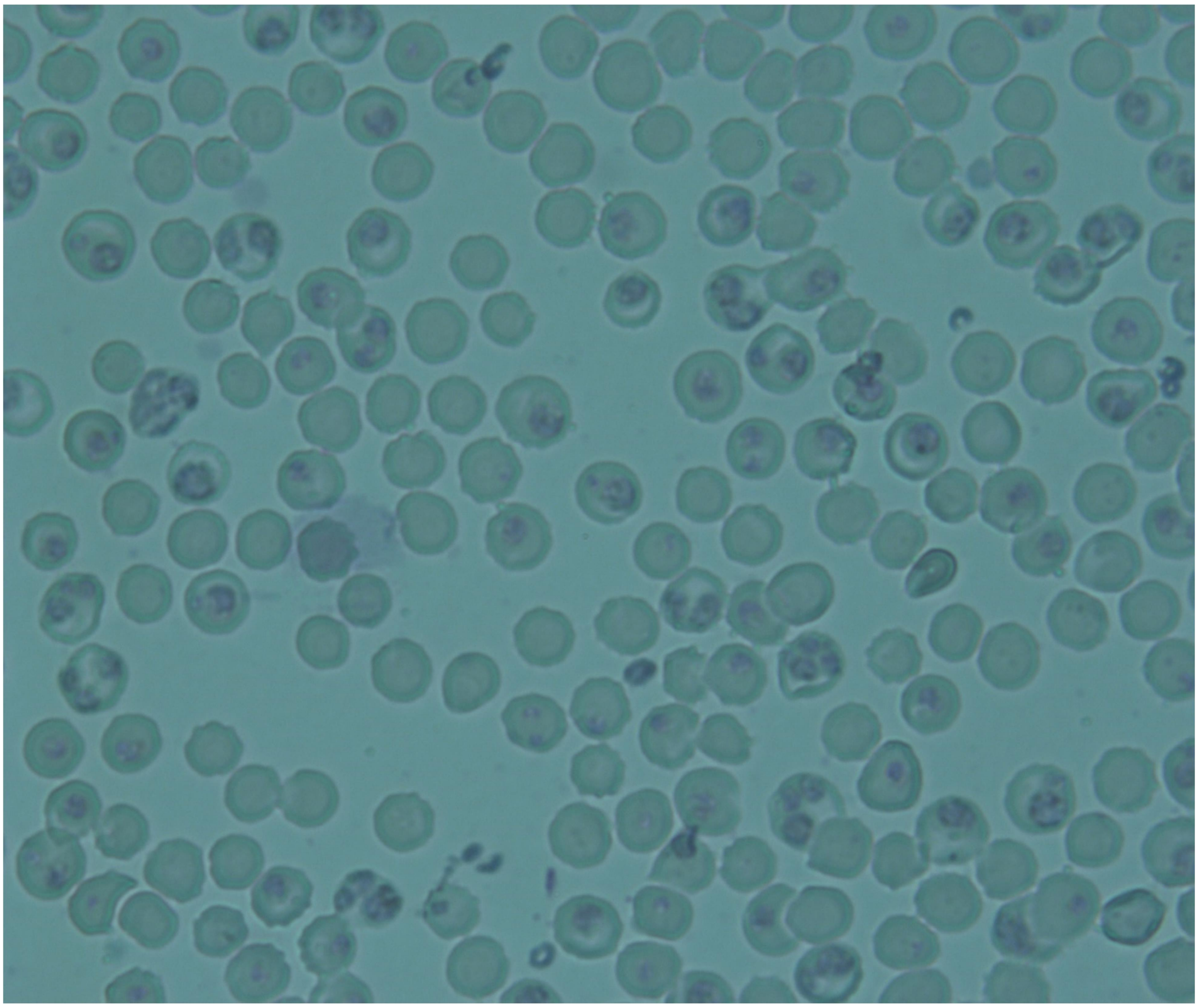

78% parasitaemia negative control in Rane’s test 2% TW80
